# Release characteristics of enoxaparin sodium-loaded polymethylmethacrylate bone cement

**DOI:** 10.1186/s13018-021-02223-w

**Published:** 2021-02-04

**Authors:** Hui Sun, Xinzhe Ma, Zhiyong Li, Jianning Liu, Wei Wang, Xiangbei Qi

**Affiliations:** 1 Department of Orthopaedic Surgery, The Third Hospital of Hebei Medical University, Shijiazhuang, China; 2 Department of Orthopaedic Surgery, Shijiazhuang Third Hospital, Shijiazhuang, China; 3 Tiemenguan District of the Third Hospital of Hebei Medical University, Tiemenguan City, China

**Keywords:** Enoxaparin sodium, Polymethylmethacrylate, Bone cement, Release characteristics

## Abstract

**Background:**

This study aimed to prepare the polymethylmethacrylate (PMMA) bone cement release system with different concentrations of enoxaparin sodium (ES) and to investigate the release characteristics of ES after loading into the PMMA bone cement.

**Methods:**

In the experimental group, 40 g Palacos®R PMMA bone cement was loaded with various amount of ES 4000, 8000, 12,000, 16,000, 20,000, and 24,000 AXaIU, respectively. The control group was not loaded with ES. Scanning electron microscopy (SEM) was used to observe the surface microstructure of the bone cement in the two groups. In the experiment group, the mold was extracted continuously with pH7.4 Tris-HCL buffer for 10 days. The extract solution was collected every day and the anti-FXa potency was measured. The experiment design and statistical analysis were conducted using a quantitative response parallel line method.

**Results:**

Under the SEM, it was observed that ES was filled in the pores of PMMA bone cement polymer structure and released from the pores after extraction. There was a burst effect of the release. The release amount of ES on the first day was 0.415, 0.858, 1.110, 1.564, 1.952, and 2.513, respectively, from the six groups with various ES loading amount of 4000, 8000, 12,000, 16,000, 20,000, and 24,000 AXaIU, all reaching the peak of release on the first day. The release decreased rapidly on the next day and entered the plateau phase on the fourth day.

**Conclusion:**

The prepared ES-PMMA bone cement has high application potential in orthopedic surgery. ES-PMMA bone cement shows good drug release characteristics. The released enoxaparin sodium has a local anti-coagulant effect within 24 h after application, but it will not be released for a long time, which is complementary to postoperative anti-coagulation therapy.

## Background

Polymethylmethacrylate (PMMA) bone cement is extensively used in various medical aspects, including antibiotic-loaded carriers, fillings for tissue defects, orthopedic implants fixation, etc. [1–3]. PMMA bone cement has been used for more than 50 years in clinical application from the initial joint prosthesis fixation to bone defect filling and vertebral body repair. The fundamental reason why PMMA bone cement can have such a wide range of applications lies in its many excellent characteristics, such as stable curing process, appropriate mechanical strength, and good biocompatibility, which cannot be replaced by other types of bone cement [4]. Moreover, PMMA bone cement is used as a matrix of drug carriers, its safety and reliability have been verified in a variety of drugs, and the antibiotic PMMA bone cement has been used in large-scale commercial applications. Produced by a beta-elimination lysis of the ordinary heparin sodium, enoxaparin sodium (ES) is the most widely used low molecular weight heparin sodium in the world, with a market share of more than 60% in the USA [5, 6]. Due to the microporous structure, PMMA bone cement as a drug carrier has been a hot topic in the interdisciplinary research of clinical medication and material sciences [7–9].

No matter what kind of drug-loaded PMMA bone cement, its characteristic is to release the drug on the site where the bone cement is used [10]. This is also the most obvious difference between ES-PMMA cement and general clinical anti-coagulation regimen in the prevention of thromboembolism. Usually, in order to prevent thrombotic pulmonary embolism, orthopedic surgeons will give subcutaneous injections of low-molecular weight heparin before major orthopedic surgery. However, this anti-coagulation is systemic and must be stopped 12 h before surgery. Then, it can be used 24 h after the surgery. This period of time is the empty window of anti-coagulation. Especially during the operation, the local coagulation system is activated, the pressure in the medullary cavity increases, the microthrombus enters the venous return, and the patient will experience cough and transient blood pressure reduction. Some studies have summarized it as bone cement implantation syndrome (BCIS). In severe cases, it can lead to suspension of surgery and even emergency rescue. ES-PMMA bone cement exerts its anti-coagulation effect in the orthopedic surgery during this empty window of anti-coagulation to reduce or avoid the occurrence of the above-mentioned situation. In this study, preliminary experiments were conducted to explore the feasibility of using PMMA bone cement as the carrier of enoxaparin sodium, which will provide a precise intervention to reduce the occurrence of pulmonary embolism during major orthopedic surgeries such as hip replacement.

## Methods

### Materials

The following equipment and materials were used in this study: standard 3D printing mold based on ISO5833:2002 “Surgical Implants-Acrylic Resin Bone Cement,” scanning electron microscope (SEM, Hebei Medical University Electron Microscopy Center, Hitachi, S-3500N), mobile C-arm X-ray machine (Siemens, Germany), vortex mixer (Shanghai INESA Co., Ltd.); digital analytical balance (Secura, Germany), vernier caliper, sterile mortar and pestle, PMMA bone cement (Palacos®R, Heraeus, Germany, 40 g/pack, batch number 88804696), ES lyophilized powder (Chengdu Baiyu Pharmaceutical Co., Ltd., 4000 AxaIU, approval number: H20150010, batch number: 16180704), ES standard (National Institutes for Food and Drug Control, 300 mg, batch number: 140810-201801), anti-thrombin III (ATIII) (10 IU/ml, Beijing Asnail Biotechnology Co., Ltd.), bovine factor Xa (FXa, 71nkat, Beijing Asnail Biotechnology Co., Ltd.), FXa chromogenic substrate S-2765 (Z-D-Arg-Gly-Arg-pNA·2HCl, 25 ml, Beijing Asnail Biotechnology Co., Ltd.), tris(hydroxymethyl) aminomethane (tris, Amresco, USA, analytical grade), ethylenediaminetetraacetic acid sodium (EDTA-2Na, International United Petroleum Chemical Co., Ltd., analytical grade), polyethylene glycol-6000 (PEG-6000, HONAM, South Korea, analytical grade), sodium chloride (Zhiyuan Chemical Reagent Co., Ltd., analytical grade), 30% acetic acid solution, and sterile deionized water.

### Preparation of ES-loaded bone cement

The temperature of the operating room was set to 23 ± 1 °C, and all experiment materials were placed in this environment for 4 h. A total of six experimental groups were set up, A to F. Various amount of ES, 4000, 8000, 12,000, 16,000, 20,000, and 24,000 AxaIU, were added respectively. Group G was the control group without ES. ES lyophilized power was grounded into powder with a mortar and then mixed thoroughly with 40 g PMMA bone cement powder. The bone cement liquid was poured (methyl methacrylate monomer) into the ES powder and mixed quickly. Following the mixing phase and waiting phase, the bone cement at the dough phase was placed in a cylindrical photosensitive resin mold with a length of 12 ± 0.1 mm and an inner diameter of 6 ± 0.1 mm. At the hardening phase, the cement was removed out of the mold carefully. A vernier caliper was used to confirm the size of the specimen to exclude the test molds that failed to meet the standards. A C-arm X-ray fluoroscopy was used to examine and to exclude the molds with obvious low density. Lastly, every test mold was weighed using a digital balance. The quality of each test mold is shown in Table 1. The preparation of ES-PMMA was completed.

**Table 1:** Weight of extracted samples and ES content in each group

Group	Sample weight (mg)	ES content (AXaIU)
A	401.2 ± 3.0	40.12
B	399.7 ± 6.5	79.94
C	398.8 ± 5.3	119.64
D	401.6 ± 4.6	160.64
E	399.2 ± 4.8	199.60
F	402.3 ± 2.7	241.38
G	400.1 ± 7.6	0

### Observation of microstructure of blank and drug-loaded PMMA bone cement

**Fig. 1:**
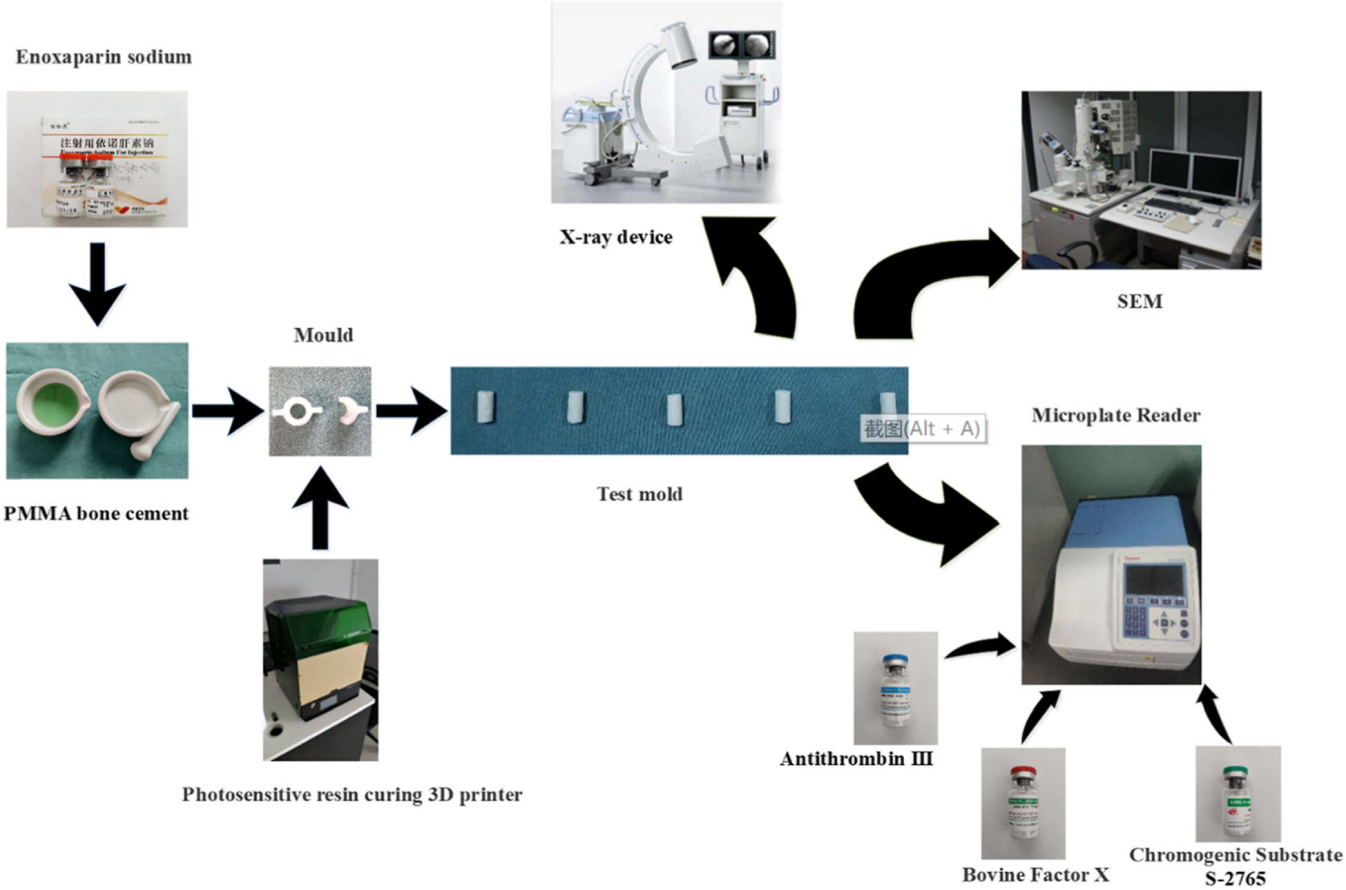
Schematic diagram of PMMA bone cement preparation and testing

One test mold from each group of ES content of 0, 8000, 16,000, and 24,000 AxaIU was analyzed by SEM. The surface of the test mold was coated with gold by a sputter coater to enhance the conductivity and the microstructure was observed by SEM (Fig. 1).

### Principle of anti-Fxa assay of ES bone cement extract

$$ \mathrm{ES}+\mathrm{ATIII}\ \left(\mathrm{excessive}\right)\to \mathrm{ES}\kern0.5em \mathrm{ATIII}\ \mathrm{compound} $$

$$ \mathrm{ES}\kern0.5em \mathrm{ATIII}+\mathrm{FXa}\left(\mathrm{excessive}\right)\to \mathrm{ES}\kern0.5em \mathrm{ATIII}\kern0.5em \mathrm{FXa}\ \mathrm{compound}+\mathrm{FXa}\left(\mathrm{residual}\right) $$

$$ \mathrm{Chromogenic}\kern0.5em \mathrm{substrate}\kern0.5em \mathrm{S}-\overset{\mathrm{FXa}\kern0.5em \left(\mathrm{residual}\right)}{2765\to \mathrm{peptide}}\kern0.5em +\kern0.5em \mathrm{p}-\mathrm{nitroaniline} $$

The peak absorption of p-nitroaniline was at 405 nm.

### Preparation of solutions

#### Tris buffer (pH 7.4)

6.06 g Tris, 2.80 g EDTA-2NA, 10.23 NaCl, and 1.00 g PEG-6000 were dissolved in 900 ml deionized water. HCl was added to adjust the pH to 7.4 and deionized water was added to make the final volume to 1000 ml. The buffer was stored at 4 °C for future use.

#### ATIII solution

Tris buffer (pH7.4) was diluted into 1 IU/ml solution.

FXa solution: Tris buffer (pH7.4) was diluted into 5nkat/ml solution.

Chromogenic substrate S-2765 solution: the storage solution was made by adding deionized water to reach a concentration of 3mmol/L and stored in a dark place in a refrigerator at 4 °C. Appropriate amount of storage solution was diluted with 5 times of volume of Tris buffer (pH7.4) into the 0.5 mmol/L working solution.

#### Standard solution

Three hundred nanograms of ES standard (30,000AXaIU) was dissolved following the instruction and diluted quantitatively with the Tris buffer (pH7.4) into solutions of four different concentrations. The concentration of solution should be within the linear range of the logarithmic dose response (generally 0.01–0.1 AxaIU per ml).

#### Extract solution

The cylinder standard test mold was labeled and placed in a 1.5 ml centrifuge tube with 1 ml sterile water. The tube was placed in a 37 °C incubator. The extract was collected every 24 h. After each extract collection, the test mold was rinsed with sterile deionized water for three times. The test mold was placed in a new centrifuge tube and added with 1 ml of sterile deionized water to continue the extraction for 10 days. The extract solution was stored at − 20 °C.

### Detection of anti-FXa potency

According to the quantitative response parallel line method in the bioassay statistical method (Chinese Pharmacopoeia 2015, Appendix 1431), the 4.4 method was used for experiment design and statistical analysis [11].

In the order of B1, S1, S2, S3, S4, T1, T2, T3, T4, T1, T2, T3, T4, S1, S2, S3, S4, and B2, 25 μl Tris pH7.4 buffer (B tube), standard solution (S tube), or extraction solution (T tube, with diluted extraction solution) was added precisely to each 1.5 ml centrifuge tube. The same volume of anti-thrombin solution was added to each tube, mixed and equilibrated at 37 °C for 2 min. Fifty microliters of factor Xa solution was then added, mixed, and equilibrated at 37 °C for 2 min. Fifty microliters of chromogenic substrate S-2765 solution was then added precisely, mixed, and equilibrated at 37 °C for 2 min. The reaction was terminated by adding 50 μl of 30% acetic acid solution. The absorbance of each tube was measured with a microplate reader at a wavelength of 405 nm. The difference in absorbance between the two tubes of blank buffer B1 and B2 must not exceed 0.05. Linear regression was depicted using the absorbance as the *y*-axis and the logarithm of the concentration of standard solution and the extraction solution as the *x*-axis, to calculate the potency and experimental error. The average confidence limit (FL%) must not be greater than 10%.

## Results

### SEM analysis of ES-PMMA bone cement (Fig. 2)

A large number of polymer beads and zirconium dioxide granules were observed under the SEM, which are the powder component of bone cement. Spherical nanoparticles are methyl methacrylate copolymers with a diameter of about 500 nm and a molecular weight of 100,000~1,000,000 g/mol or higher. The spherical particles are tightly connected to each other, and the irregular substance between them is solidified enoxaparin sodium. As ES is gradually added to PMMA bone cement, the gaps of the copolymer microspheres of bone cement are gradually filled with ES, and this filling has little effect on the original structure of PMMA bone cement. In other words, adding ES did not significantly affect the polymer structure of the bone cement.

**Fig. 2:**
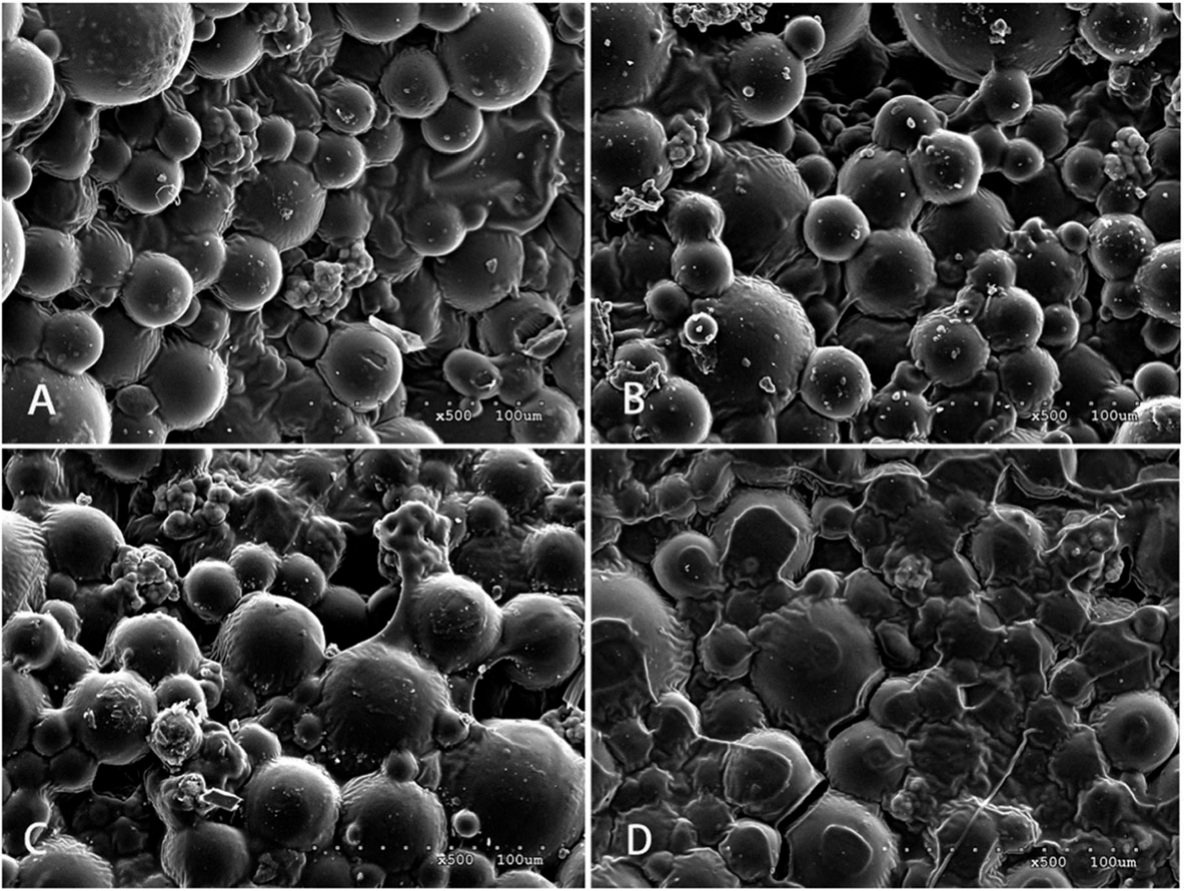
SME image of ES-PMMA bone cement. **a** No ES loaded PMMA bone cement. **b** 40 g PMMA bone cement + 8000 AxaIU ES. **c** 40 g PMMA bone cement + 16,000 AxaIU ES. **d** 40 g PMMA bone cement + 24,000 AxaIU ES

The pores between the beads are the carriers and space for drug release. The PMMA bone cement absorbs water through these pores and releases the water-soluble drugs by dispersion [12, 13]. ES is a mucopolysaccharide substance with a relative molecular mass (Mr) of 3500–5500 with good water solubility. With the increased amount of ES added in the PMMA bone cement, the pores between the polymers were filled. Under the SEM, the beads became shallow and some sugar coating-like substances were observed. Therefore, the ES-PMMA bone cement was demonstrated be the ES carriers.

### Release of ES-PMMA bone cement in vitro

The anti-FXa potency of bone cement extract solution loaded with various amount of EX is shown in Table 2. The curve was depicted using the anti-FXa potency of extract solution as y-axis and the measurement days as x-axis.

**Table 2:** The potency of ES release at various timepoint by ES-PMMA with different ES loading amount

ES loading amount(IU)	Potency of extraction solution (IU/ml)									
	1 day	2 days	3 days	4 days	5 days	6 days	7 days	8 days	9 days	10 days
4000	0.415	0.078	0.066	0.047	0.059	0.043	0.046	0.040	0.042	0.037
8000	0.858	0.285	0.146	0.037	0.029	0.035	0.031	0.029	0.028	0.025
12,000	1.110	0.314	0.214	0.091	0.095	0.088	0.085	0.082	0.081	0.074
16,000	1.564	0.376	0.205	0.051	0.065	0.062	0.058	0.055	0.056	0.046
20,000	1.952	0.538	0.280	0.060	0.082	0.068	0.073	0.079	0.054	0.063
24,000	2.513	0.953	0.351	0.148	0.071	0.075	0.093	0.060	0.082	0.076

**Fig. 3:**
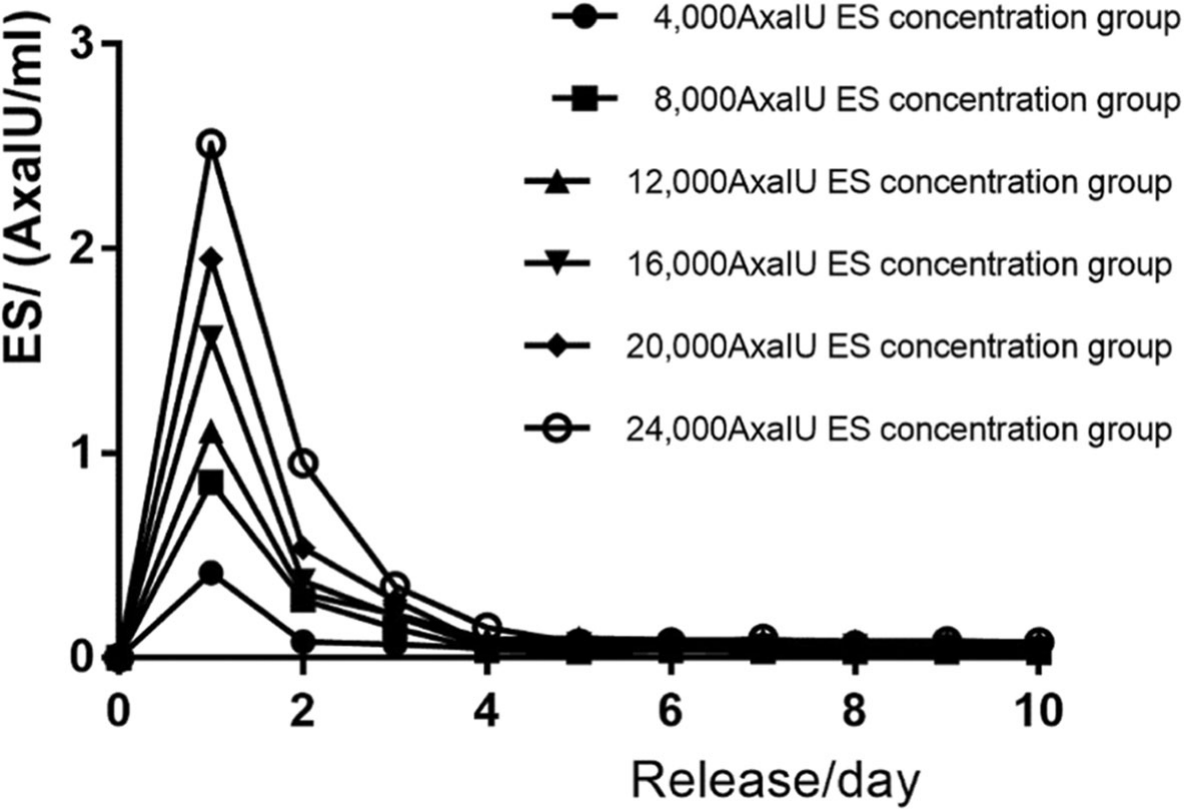
In vitro release curve of ES-PMMA bone cement

As shown in Fig. 3, the bone cement systems with various ES loading amount all released ES fast in the beginning and there was a burst release effect. The peak ES release occurred on the first day and then the release rapidly decreased. Plateau was shown from day 4 when the release rate became stable.

## Discussion

### Application of ES-loaded PMMA bone cement

It has been over 90 years since the discovery of heparin as an effective anti-coagulant. The mechanism of anti-coagulant is to activate anti-thrombin III (ATIII) by the allosteric effect through the specific combination of polysaccharide sequence and ATIII [14–16]. The activated ATIII can inhibit a variety of coagulation factors including Xa, IIa, IXa, XIa, XIIa etc., to exert the anti-coagulant activity. Researchers have been working on the improvement of heparin to overcome some of the inherent shortcomings, such as short biological half-life, risk of bleeding, and the need of blood coagulation monitoring. Enoxaparin is a low molecular weight heparin produced using the ordinary heparin as the raw material and obtained by β-elimination degradation. Compared with ordinary heparin, enoxaparin has a strong anti-thrombotic effect, weak anti-coagulant effect, low incidence of side effects such as bleeding, good absorption of subcutaneous injection, long biological half-life, and predictable anti-coagulant effect. At present, enoxaparin has replaced the ordinary heparin in clinical practice [17–20]. Enoxaparin sodium has an average molecular weight of 3800–5000, anti-FXa activity of 90–125 IU/mg, anti-FIIa activity of 20–35 IU/mg, anti-FXa/anti-FIIa ratio of 3.3–5.3, and an excellent water solubility [21].

### Principles of PMMA bone cement as drug carriers

**Fig. 4:**
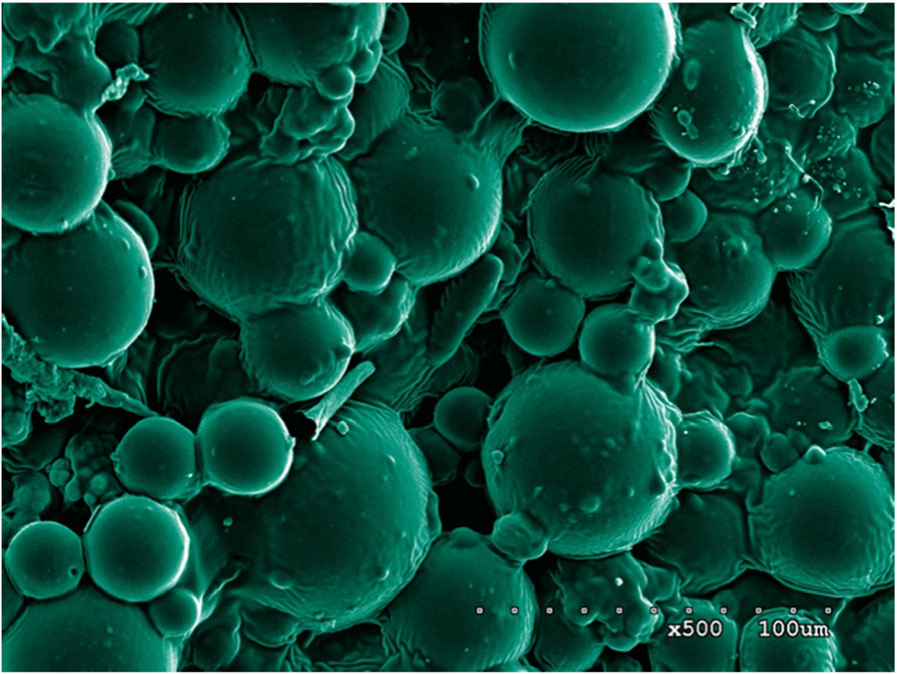
A large number of polymer beads and zirconium dioxide granules were observed under the SEM, which are the powder component of bone cement. The pores among the beads are the carriers and space for drug release. The PMMA bone cement absorbs water through these pores and releases the water-soluble drugs by dispersion

PMMA bone cement has been widely used in clinical practice, such as total hip replacement, half hip replacement, total knee replacement or single patella replacement, vertebroplasty, bone tumors, pathological fractures, spacers with PMMA bead chain during infection, or placeholders for soft tissue defect in open fracture, etc. [22–26]. As the drug carrier, the most mature and largest-scale commercial application of PMMA bone cement is antibiotic-loaded PMMA bone cement [27–30]. From the experience of antibiotic-loaded bone cement, certain physical and chemical properties are required to facilitate the effective release of drug from bone cement and act on the body. These properties include high water solubility, resistance to radiation disinfection or epoxy ethane sterilization, stability when stored with bone cement powder before use, inactivity during bone cement polymerization, high temperature resistance, little or no effect on mechanical strength of bone cement, and good release from solid bone cement [29, 31–35]. Stevens et al. [36] reported that the characteristics of drug release varied a lot when using different brands of bone cement. The best release characteristics were tested from Palacos®R bone cement. Another bone cement Simplex®, which has been widely used in clinical practice, exhibited poor release characteristics. The bone cement with good release characteristics may have more pores and looser structure among the polymer beads after polymerization, thus more amount of drug can be loaded and the contact surface with solution is larger. Based on the above reasons, the Palacos®R bone cement was selected as the ES carrier in this study (Fig. 4).

**Fig. 5:**
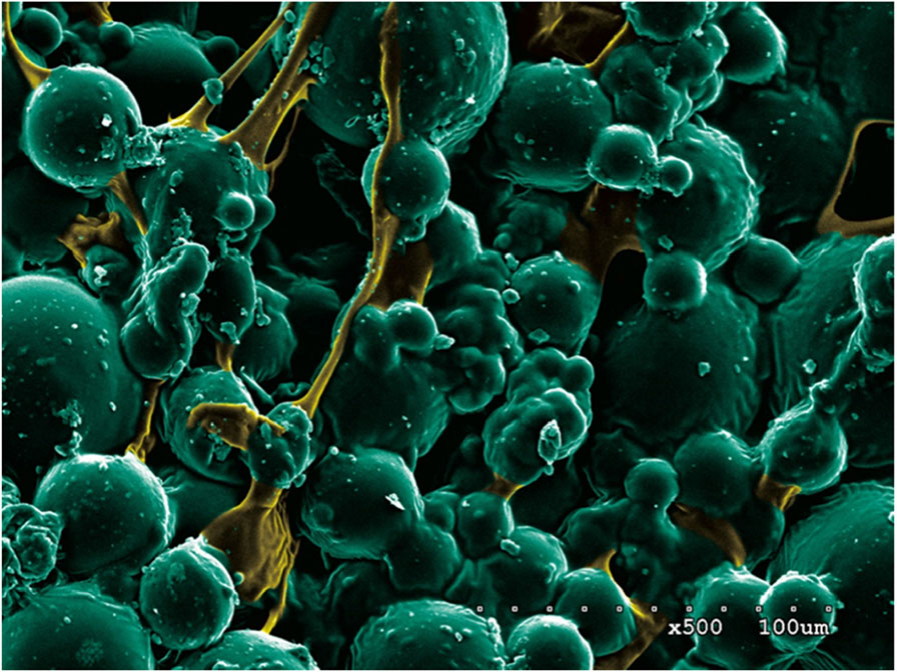
Enoxaparin sodium is a mucopolysaccharide substance with a relative molecular weight (Mr) of 3500–5500 with good water solubility. With the increase amount of ES added in PMMA bone cement, the pores between the polymers were filled and the gaps between beads became shallow. Some sugar-like substances were also observed under the SEM. Therefore, ES-PMMA bone cement is considered as a carrier which can release enoxaparin sodium.

The surface of no ES-loaded bone cement was observed under the SEM. Most of the beads observed were large and uneven copolymers, which are the polymethyl methacrylate copolymers manufactured by industrial grinding. These beads were bound together by PMMA during the polymerization reaction of methyl methacrylate (MMA). There were pores of various sizes between the polymer beads. The entire PMMA bone cement formed a sponge-like three-dimensional structure which serves as the structural basis for drug loading (Fig. 5).

**Fig. 6:**
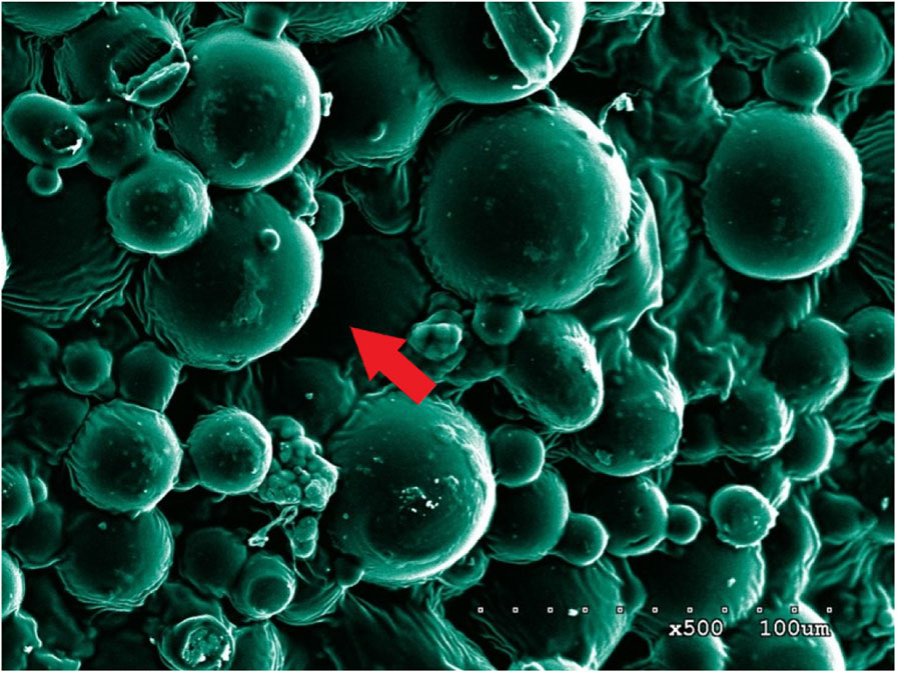
Ten days after extraction, the samples with the same loading amount were scanned again under SEM. It was observed that the filamentous material between the beads disappeared and pore size between the beads was increased

Syrup-like substances between the copolymer beads were observed on ES-loaded PMMA bone cement. With increased ES loading amount, the syrup-like substances also increased and wrapped on the surface of copolymers beads like sugar coating. Comparison between the SEM photos before and after drug release revealed that the syrup-like substances were reduced significantly. We inferred that these substances were ES, a highly sulfated glycosaminoglycan. After drug release the pore space became large. The super large pores with diameters > 500 um were observed in multiple SEM photos. By providing channels for capillary growth and an environment and framework suitable for cell growth, these pores enhanced the biocompatibility of drug-loaded bone cement and mechanical strength in connection with the surrounding tissues [37, 38] (Fig. 6).

### Mixing process of ES-PMMA bone cement

There are two mixing methods in the drug-bone cement release system. One is to thoroughly and evenly mix the drug lyophilized powder or fine particles with bone cement powder component. The liquid component is poured into the mixture before use and evenly mixed vacuumed or manually. The other is to thoroughly and evenly mix the drug lyophilized powder or fine particles with bone cement powder component. The drug is added in the dough phase so that the drug is wrapped within the bone cement dough [39, 40]. The first method is used in various antibiotic-loaded bone cement on the market, such as Palacos®R+G and CMW®1G. These mature products are all prepared using this way in order to make the drug component evenly distributed inside the bone cement and make the product more convenient to use [41, 42]. The second method is more used in the operating room and prepared manually. The advantages include various amount of drug loading and specific individual treatment. However, the disadvantages are obvious. The uneven mixing has a great impact on bone cement mechanical strength, and uneven drug distribution makes it hard to predict the release characteristics [43–45]. In order to minimize the influence of human factors on the release characteristics of bone cement, the first mixing method was used. The ES lyophilized powder was fully ground into fine particles, mixed with bone cement powder thoroughly until no drug particles are visible to the naked eye and then mixed with the liquid components of bone cement to make the test mold for the experiment.

### ES-PMMA bone cement release characteristics

The release mechanism of the drug-bone cement release system follows the principle of dispersion. Dry bone cement absorbs water, and water-soluble drugs are released with the random and irregular thermal motion of water molecules [46]. The factors which affect the drug release rate from the bone cement include (1) absorption of water, surface area, and porosity of bone cement, (2) nature and content of drug, and (3) mixing method [47–49]. Faster water absorption, larger surface area and higher porosity lead to a higher drug release rate and release amount. Regarding the effect of drug nature and content on drug-loaded bone cement release system, Kuehn et al. [50] reported that the particle size of drug affects the release amount under the comparable conditions of PMMA bone matrix, operating separation, mixing techniques, and drug content. The release amount of coarse drug particles is higher than that of fine drug articles, which is higher than that of very fine drug articles. The drug release amount of manual mixing is greater than that of vacuum mixing when the other conditions are comparable. This is because vacuum mixing may decrease the porosity of PMMA bone cement, and drug is released from the pores of bone cement matrix. With less pores, drug release is decreased [51, 52]. Despite careful grinding of the ES lyophilized powder in the mortar in the experiment, the drug particles were very coarse while the bone cement powder particles were very fine. There was visible difference between the two, and it was not obvious until the two particles were completed mixed. The liquid bone cement was mixed carefully with the powder mixture for 30 s manually following the user instruction of Palacos®R bone cement to obtain the ES-loaded bone cement for experimental use. The test mold prepared in this way exerted higher release amount and a faster release rate than the product manufactured in ideal conditions (prechilled super fine particles of ES and bone cement powder mixed under vacuum).

**Fig. 7:**
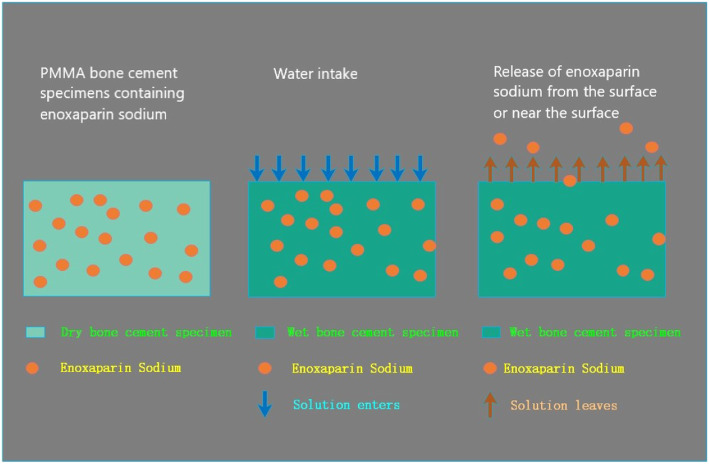
Schematic diagram of the ES-PMMA bone cement release theory

The release mechanism of the drug-bone cement release system follows the principle of dispersion (Fig. 7). Dry bone cement absorbs water, and water-soluble drugs are released with the random and irregular thermal motion of water molecules. Due to its good water solubility, ES is easily released into the surrounding tissues with the water.

**Fig. 8:**
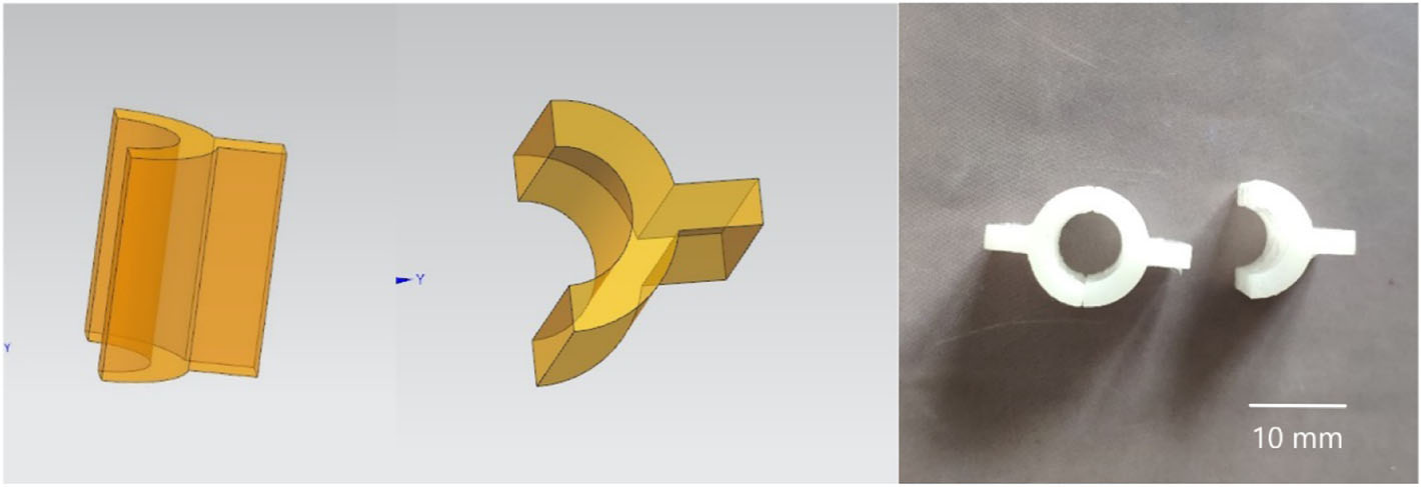
CAD mold design and mold made by 3D printing

In order to produce accurate column bone cement test mold with a length of 12 mm and a diameter of 6 mm, we adopted 3D printing technology (Fig. 8). First, draw the design drawing of the mold with Auto CAD software. After importing the design drawing into 3D printer, the machine prints the mold with high polymer resin. The white mold in Fig. 8 is the finished product. It is characterized by a smooth surface, which can be closed and separated when used, and it is very convenient to take out the solidified bone cement.

The mold used for the preparation of the test mold was made according to ISO5833:2002 “Surgical Implant—Acrylic Resin Bone Cement”. Computer-aided design was used for mold drawing and mold was made by 3D printing using photosensitive resin. The test mold was a cylinder with a length of 12 ± 0.1 mm and a diameter of 6 ± 0.1 mm, with regular shape and smooth surface, as shown in Fig. 8. This test mold is quite different with the shape of bone cement implanted in human body in clinical practice. In the actual clinical application, the solidified bone cement closely adheres to the inner wall of the medullary cavity or the bone surface, with an irregular shape and rough surface. These features increase the surface area of bone cement [53, 54]. Riva et al. [55] found that most of the drug in the drug-bone cement release system cannot be released. The drug is released from a thin layer on the bone cement surface. Release amount is proportional to the surface area of the bone cement. Thus, the larger surface area per unit volume of bone cement, the more the drug is release. Therefore, we believe that the release amount from the actual drug loaded bone cement system implanted in the human body is greater than that of the in vitro release system.

**Fig. 9:**
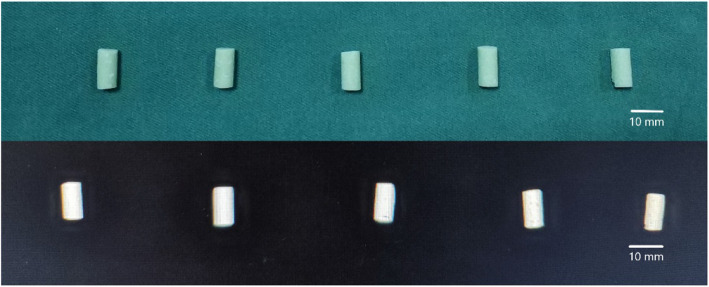
Bone cement test mold prepared by the mold

Each bone cement column test mold has undergone X-ray inspection, and any one with uneven density will be excluded from the experiment (Fig. 9).

### The release effect of ES-PMMA bone cement

The absorbance value of extract of ES bone cement can be obtained by the chromogenic substrate method. The value was converted into a logarithm. According to the quantitative response parallel line method documented in Chinese Pharmacopoeia 2015, Appendix 1431, the 4.4 method was used to calculate the potency and experimental error and to depict the release curve. The curve showed that the ES was released at a high concentration and reached the peak on the first day. The release then rapidly decreased to a low range and became stable. Consistent with the results reported by Anguita-Alonso et al. [56], our results showed that the drug-bone cement release system generally has a burst effect and the sustained release may last for a long time. The anti-FXa activity for the therapeutic effect of ES to prevent thrombosis is 0.20–0.50 AxaIU/ml and may reach 1.0 AxaIU/ml under the therapeutic amount. In the experiment with 4000 AxaIU ES added to 40 g PMMA bone cement, the release amount reached the therapeutic dose (about 0.40 AxaIU/ml) within 24 h. With 8000 AxaIU ES loaded to 40 g PMMA bone cement, the release amount reached the therapeutic dose (about 0.40 AxaIU/ml) within 24 h and drug concentration maintained at the range between the preventive dose and therapeutic dose. With addition of 8000 AxaIU or more of ES, the drug centration released in 24 h exceeded the maximum therapeutic dose of anti-coagulation, which may cause a bleeding risk. Therefore, it is recommended to choose the ES amount within a safe drug concentration.

## Conclusion

Enoxaparin can be physically embedded into the pore structure of PMMA bone cement. Enoxaparin sodium can be released from the solidified PMMA bone cement. Enoxaparin does not participate in the polymerization of PMMA bone cement and can tolerate the heat released by polymerization, with its own anti-coagulant activity unchanged.

## Data Availability

Not applicable.

## Abbreviations

PMMA: Polymethylmethacrylate
ES: Enoxaparin sodium
SEM: Scanning electron microscopy
Tris-HCl: Tris(hydroxymethyl)aminomethane
FXa: Factor Xa
AXaIU: Anti-Xa international unit
